# Epithelial-To-Mesenchymal Transition Markers and CD44 Isoforms Are Differently Expressed in 2D and 3D Cell Cultures of Prostate Cancer Cells

**DOI:** 10.3390/cells8020143

**Published:** 2019-02-11

**Authors:** Fabrizio Fontana, Michela Raimondi, Monica Marzagalli, Michele Sommariva, Patrizia Limonta, Nicoletta Gagliano

**Affiliations:** 1 Department of Excellence: Department of Pharmacological and Biomolecular Sciences, Università degli Studi di Milano, via Balzaretti 9, 20133 Milan, Italy; fabrizio.fontana@unimi.it (F.F.); michela.raimondi@unimi.it (M.R.); monica.marzagalli@unimi.it (M.M.); patrizia.limonta@unimi.it (P.L.); 2 Department of Biomedical Sciences for Health, Università degli Studi di Milano, via Mangiagalli 31, 20133 Milan, Italy; michele.sommariva@unimi.it

**Keywords:** epithelial-to-mesenchymal transition, 3D-spheroids, E-cadherin, CD44, prostate cancer

## Abstract

Three-dimensional (3D) cell cultures allow the mimic of functions of living tissues and provide key information encoded in tissue architecture. Considered the pivotal role of epithelial-to-mesenchymal transition (EMT) in carcinoma progression, including prostate cancer (PCa), we aimed at investigating the effect of the 3D arrangement on the expression of some key markers of EMT in cultured human prostate cancer (PCa) cells, to better understand PCa cell behavior. PC3 and DU145 PCa cells were cultured in RPMI cell culture medium either in 2D-monolayers or in 3D-spheroids. The main EMT markers E-cadherin, N-cadherin, α-smooth muscle actin (αSMA), vimentin, Snail, Slug, Twist and Zeb1 were evaluated by confocal microscopy, real-time PCR and Western blot. Confocal microscopy revealed that E-cadherin was similarly expressed at the cell boundaries on the plasma membrane of PCa cells grown in 2D-monolayers, as well as in 3D-spheroids, but resulted up-regulated in 3D-spheroids, compared to 2D-monolayers, at the mRNA and protein level. Moreover, markers of the mesenchymal phenotype were expressed at very low levels in 3D-spheroids, suggesting important differences in the phenotype of PCa cells grown in 3D-spheroids or in 2D-monolayers. Considered as a whole, our findings contribute to a clarification of the role of EMT in PCa and confirm that a 3D cell culture model could provide deeper insight into the understanding of the biology of PCa.

## 1. Introduction

Prostate cancer (PCa) is a urological disease associated with significant morbidity and mortality, representing the second most common cause of cancer-related deaths in male population [1].

There are considerable data suggesting that epithelial-to-mesenchymal transition (EMT) plays a pivotal role in PCa progression [2]. An EMT program is required by cancer cells to acquire functional characteristics for metastasis, and involves multiple steps including the acquisition of invasiveness, intravasation in systemic blood or lymphatic systems, subsequent extravasation, and growth at distant organs [3]. During EMT, cancer cells shed their epithelial features, detach from epithelial sheets, loose their polarity, and undergo cytoskeletal modifications towards a mesenchymal phenotype, acquiring a high degree of motility and invasive potential [4]. The EMT-related phenotype is characterized by E-cadherin down-regulation, expression of vimentin and α-smooth muscle actin (αSMA), and secretion of matrix metalloproteinases (MMPs) [4].

Several inducers of EMT acting as transcription factors have been described, such as Snail, Slug, Twist and Zeb. They repress the expression of E-cadherin and induce the expression of mesenchymal genes, like vimentin and N-cadherin [5,6,7]. Repression of E-cadherin expression by EMT transcription factors was described in vivo and in various cancer cell lines, including lung, breast, colorectal and ovarian cancer, thus inducing tumor malignancy [8,9,10]. 

E-cadherin down-regulation is considered a key event in the EMT process. E-cadherin loss or decrease at the cell membrane of cancer cells has often been associated with worsening histological grade and clinical stage, along with poor prognosis in a variety of tumors, including breast, pancreatic, gastric, and prostate cancer [5,11,12,13,14].

However, some heterogeneity in E-cadherin expression has been described in PCa, showing variable E-cadherin levels in metastatic tissues compared to primary tumor tissues [15,16,17,18,19,20].

Recently, EMT has been linked to stemness, since cells undergoing EMT acquire stem cell-like features [21,22]. Stem-like cells acquire a more complete EMT molecular profile, and are reported to express the cancer stem/progenitor cells marker CD44, which plays an important role in inducing EMT and/or in maintaining the mesenchymal phenotype in PCa [23,24]. Moreover, both downregulation of CD44 standard splice isoform (CD44s) and dysregulated splicing were described, leading to increased, aberrant CD44 variant splice isoforms (CD44v) that play a role in tumor invasion and as prognostic markers. Both CD44s and CD44v have been linked to the cancer stem cell (CSC) niche and, therefore, to cancer progression [25,26].

It is generally recognized that cancer cell phenotype can be characterized in vitro in three-dimensional (3D) cell culture systems, reducing the gap between 2D cell cultures and physiological tissues. Maintaining a 3D cell arrangement that reflects the in vivo situation in tissue and tumors in relation to cell-cell interaction and differentiation patterns offers the possibility to better analyze cancer cell biology and behavior [27,28,29,30,31,32]. 3D cultures models were also used to investigate cell-cell interactions in prostate carcinoma cells [33,34].

Since 3D spatial organization addresses the complex interplay of cell-cell interaction and 3D cultures mimic the 3D structure of living tissue, the aim of this study was to investigate the expression of the main EMT markers in PCa cell lines (PC3 and DU145), the most common type of human castration resistant prostate cancer cells employed in experimental in vitro studies, grown in either 2D-monolayers or in 3D-spheroids, in order to better investigate their phenotype and, therefore, their behavior. Moreover, since EMT has been linked to stem cell phenotype [35,36,37], we also analyzed the expression of CD44 isoforms in the same experimental conditions. Our data show that PCa cells grown in 3D spheroids compared to 2D monolayers are characterized by a different expression of some key EMT markers and, for the first time to the best of our knowledge, of CD44s and variants, contributing to new knowledge of the role of EMT markers, particularly E-cadherin, in PCa cell biology.

## 2. Materials and Methods

### 2.1. 2D-Monolayer Cell Culture and 3D-Spheroid Preparation

PC3 and DU145 (American Type Culture Collection, ATCC) human castration-resistant prostate cancer cell lines were cultured in 2D and 3D cell cultures. PC3 and DU145 cell lines were chosen, since they are the most common and well characterized type of human castration resistant prostate cancer cells employed in in vitro experimental studies. PC3 and DU145 cells were cultured in RPMI cell culture medium, supplemented with 7.5% (PC3 cells) or 5% (DU145 cells) heat-inactivated fetal bovine serum (FBS), 2 mM glutamine, antibiotics (100 U/mL penicillin, 0.1 mg/mL streptomycin), and cell viability was determined by trypan blue staining. To obtain 3D-spheroids, PCa cells (5 × 10^4^ cells) were seeded in 24-well multi-well plates coated with 1% agarose in serum-free cell culture medium. Spheroid integrity was verified by phase-contrast imaging after 3 days, one week and two weeks of culture. Since spheroid morphology, integrity, and cell adhesion were not modified starting from day 10 and thereafter, spheroids were harvested after 10 days for morphological and molecular evaluations. Approximately 300 spheroids were cultured, having a mean 500 µm or 1 mm diameter, respectively, for DU145 and PC3 3D cell cultures. For molecular analysis, spheroids were pooled and harvested into two separate pools to obtain duplicate samples of PC3 and DU145. Duplicate samples of the same cells grown in 2D-monolayers were analyzed when at confluence.

### 2.2. Immunofluorescence and Confocal Microscopy 

For 2D-monolayers analysis, PC3 and DU145 were cultured on 12-mm diameter round coverslips into 24-well culture plates. When at confluence, cells were washed in phosphate-buffered saline (PBS), fixed for 10 min at room temperature in 4% paraformaldehyde in PBS containing 2% sucrose, post-fixed in 70% ethanol and stored at −20 °C until use. 3D-spheroids were fixed for 3 h in the same conditions. 3D-spheroids were processed “free-floating” in the wells of 24-well culture plates during fixation and immunofluorescence procedures. Cells grown in 2D-monolayers and 3D-spheroids were then washed in PBS three times and incubated for 2 h with the primary antibodies anti-Ki-67 (1:100, Dako, Agilent, Santa Clara, CA, USA), anti-E-cadherin (1:2500, Becton Dickinson, Milan, Italy), anti-N-cadherin (1:200, Santa Cruz Biotechnology Inc., Dallas, TX, USA), anti-vimentin (1:200, Novocastra, Leica Microsystems, Milan, Italy), anti- αSMA (1:400, Sigma-Aldrich, Milan, Italy). Secondary antibodies conjugated with Alexa 488 (1:500, Molecular Probes, Invitrogen Life Technologies, Monza, Italy) were applied for 1 h at room temperature in PBS in the dark. Negative controls were incubated omitting the primary antibody. Finally, cells on coverslips and 3D-spheroids were incubated for 15 min with 4′,6-diamidino-2-phenylindole (DAPI) (1:100.000, Sigma-Aldrich) and mounted onto glass slides using Mowiol (Sigma-Aldrich). 

Sample imaging of PCa cells grown in 2D-monolayers or in 3D-spheroids was performed using a laser scanning confocal microscope Leica TCS SP8 X (Leica Microsystems GmbH, Mannheim, Germany). The fluorochromes were excited by a continuous wave 405 nm diode laser and a pulsed super continuum White Light Laser (470–670 nm; 1 nm tuning step size). DAPI was excited using diode laser and detected from 409 to 507 nm, AlexaFluor-488 was excited selecting 499 nm-laser line and detected from 504 to 647 nm. The images were acquired in the scan format 1024 × 1024 pixel using a HC PL APO 40×/1.30 CS2 oil immersion objective and a pinhole set to 1 Airy unit. Data were acquired using the Leica LAS X rel. 3.1.1.15751 software (Leica Microsystems GmbH, Mannheim, Germany).

### 2.3. Real-Time PCR 

Total RNA was isolated by a modification of the acid guanidinium thiocyanate-phenol-chloroform method (Tri-Reagent, Sigma-Aldrich). For each RNA extraction, approximately 70 3D-spheroids or 3D aggregates and 10–15 × 10^6^ confluent monolayer cells were used. One µg of total RNA was reverse-transcribed in 20 µL final volume of reaction mix (Bio-Rad, Milan, Italy). mRNA levels for E-cadherin, N-cadherin, Snail, Slug, Twist, Zeb1, CD44s and variants were assessed. Glyceraldehyde 3-phosphate dehydrogenase (GAPDH) was used as internal control to normalize the differences in the amount of total RNA in each sample. Primers used for real time PCR are listed in Table 1.

Amplification reactions were conducted in a 96-well plate in a final volume of 20 µL per well, containing 10 µL of 1× SYBR Green Supermix (Bio-Rad), 2 µL of template, 300 pmol of each primer, and each sample was analyzed in triplicate in a Bioer LineGene 9600 (Bioer, Hangzhou, China). The cycle threshold (Ct) was determined and gene expression levels relative to that of GAPDH were calculated by the 2^−ΔCt^ method. 

### 2.4. Western Blot 

Cell lysates were prepared in RIPA buffer, incubated on ice for 30 min and centrifuged at 14,000× *g* for 15 min at 4 °C to remove cell debris. Cell lysates (20 μg of total proteins) were diluted in sample buffer (Bio-Rad), separated by SDS-PAGE under reducing and denaturing conditions and transferred onto nitrocellulose membranes. After blocking, membranes were incubated with the primary antibodies against E-cadherin (1:2500, Becton Dickinson, Milan, Italy), N-cadherin (1:1000, Cell Signaling Technology Inc., Danvers, MA, USA), Vimentin (1:1000, Leica-Microsystems, Milan, Italy), Snail (1:1000, Cell Signaling Technology Inc.), Slug (1:1000, Cell Signaling Technology Inc.), Twist (1:1000, Cell Signaling Technology Inc.) and Zeb1 (1:1000, Cell Signaling Technology Inc.). Detection was done using horseradish peroxidase-conjugated secondary antibodies (Cell Signaling Technology Inc.) and enhanced chemiluminescence Westar Eta C Ultra 2.0 reagents (Cyanagen, Bologna, Italy). To confirm equal loading, membranes were reprobed with α-tubulin (1:2000, Sigma-Aldrich).

### 2.5. Statistical Analysis

Data are expressed as mean ± SD. Comparison between 2D-monolayers and 3D-spheroids were calculated using independent samples two-tailed *t* test. *p* values lower than 0.05 were considered significant.

## 3. Results

### 3.1. 2D-Monolayer and 3D-Spheroid Morphology 

**Figure 1:**
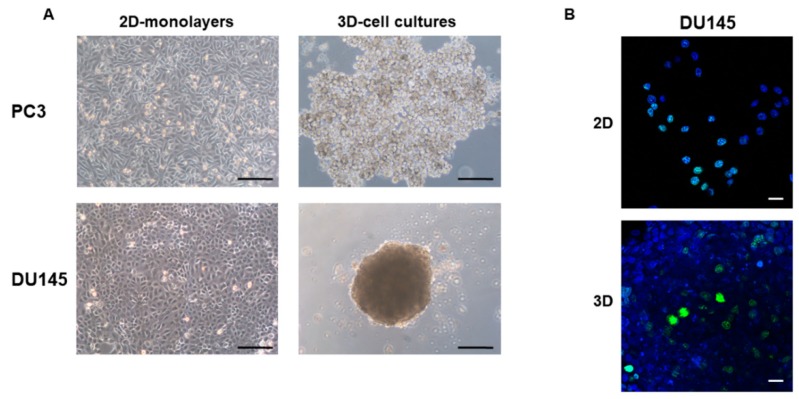
Morphology of prostate cancer (PCa) cells grown in 2D-monolayers and 3D cell cultures. (**A**) Micrograph at the inverted microscope showing the epithelial morphology of PC3 and DU145 cells grown in 2D-monolayers and 3D cell cultures after 10 days. Original magnification: 10×. (**B**) Confocal microscopy showing Ki-67 expression in DU145 grown in 2D-monolayer and 3D-spheroid. Original magnification: 40×. Blue: DAPI; green: Ki-67. Bar: 200 µm (**A**), 20 µm (**B**).

PC3 and DU145 PCa cells cultured in 2D-monolayers displayed a polygonal morphology with tightly apposed cells, consistent with an epithelial phenotype (Figure 1A). When seeded in agarose-coated wells, PC3 and DU145 PCa cells formed 3D aggregates and 3D-spheroids, respectively, evident after 40–72 h. 3D cell cultures containing PC3 cells exhibited an irregular morphology and cells were less densely apposed. In contrast, spheroids containing DU145 cells had a spheroidal regular morphology and they contained densely packed and strongly adhering cells, as previously described [33] (Figure 1A). Since PC3 3D-aggregates did not maintain their integrity during manipulation, immunofluorescence analysis was performed only on DU145 3D-spheroids.

To demonstrate that 3D-spheroids are not just an aggregate of apposed cells, but that they represent a 3D-cell culture, they were incubated with Ki-67 antibody to detect cell proliferation. Ki-67 protein is a proliferation marker detectable during all active phases of the cell cycle (G(1), S, G(2), and mitosis), but absent in resting cells (G(0)) [37]. We observed proliferating cells in both 2D-monolayers and homogeneously throughout 3D-spheroids containing DU145 cells (Figure 1B), confirming that cells cultured in 3D-spheroids maintain their proliferative phenotype. Moreover, the homogeneous distribution of proliferative cells in 3D-spheroids allows one to exclude the idea that the eventual different expression of EMT markers in different regions of the spheroids is not a consequence of a different proliferation phenotype. 

### 3.2. E-Cadherin Expression 

**Figure 2:**
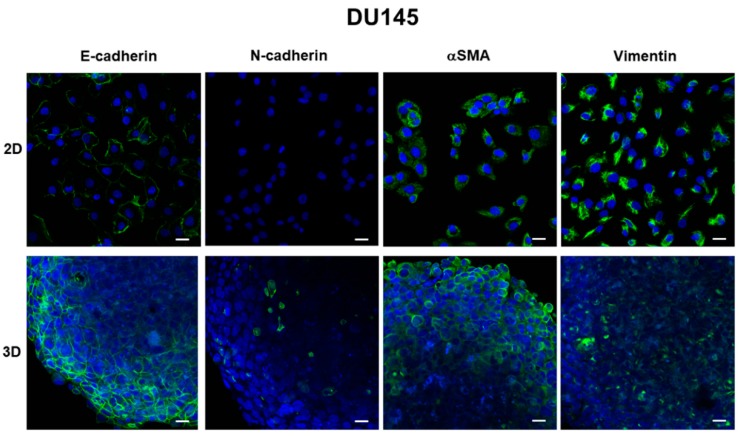
Immunofluorescence analysis of epithelial-to-mesenchymal (EMT) markers in DU145 cells. Micrographs using the confocal microscope showing the epithelial marker E-cadherin and mesenchymal markers N-cadherin, αSMA and vimentin (green) in DU145 cells grown in 2D-monolayers and in 3D-spheroids. Original magnification: 40×. Bar: 20 µm. Blue: DAPI.

**Figure 3:**
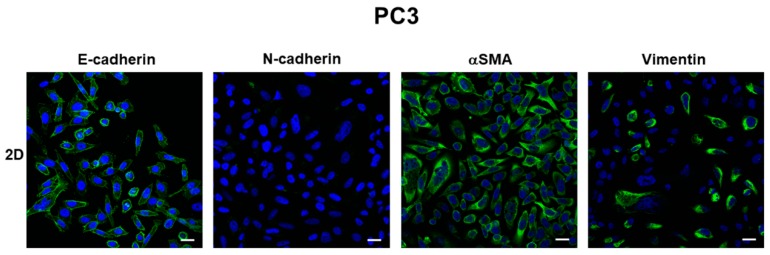
Immunofluorescence analysis of epithelial-to-mesenchymal transition (EMT) markers in PC3 cells. Micrographs using the confocal microscope showing the epithelial marker E-cadherin and mesenchymal markers N-cadherin, αSMA and vimentin (green) in PC3 cells grown in 2D-monolayers. Original magnification: 40×. Bar: 20 µm. Blue: DAPI.

Immunofluorescence analysis revealed that E-cadherin was expressed at cell boundaries in both DU145 and PC3 2D-monolayers. A similar expression was observed in DU145 3D-spheroids, consistent with the presence of functional adherens junctions, but E-cadherin immunoreactivity was more evident in the peripheral region of the spheroids (Figure 2, Figure 3 and Figure S1). 

**Figure 4:**
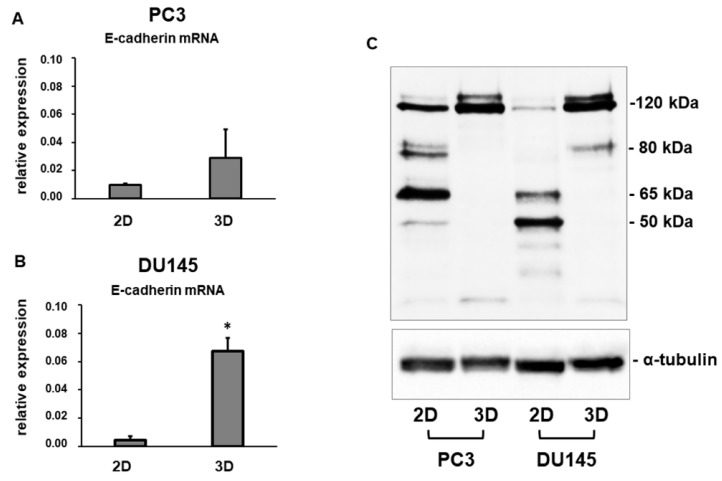
E-cadherin mRNA and protein levels. Bar graphs showing E-cadherin mRNA levels in PC3 cells (**A**) and DU145 cells (**B**) grown in 2D-monolayers and 3D cell cultures. Data are means ± SD of duplicate samples. (**C**) Representative Western blot analysis showing E-cadherin protein levels in whole cell lysates obtained from PC3 and DU145 cells cultured in both experimental conditions. * *p* < 0.05 vs DU145 2D-monolayer.

Gene expression analysis revealed that E-cadherin mRNA levels were expressed at a lower extent in PC3 and DU145 cells grown in 2D-monolayers compared to 3D-cell cultures, and that E-cadherin mRNA was up-regulated in PC3 3D aggregates (*p* ns) and DU145 3D-spheroids (*p* < 0.05), compared to 2D-monolayers (Figure 4A,B). Western blot analysis confirmed this expression pattern, showing that the gene expression pattern was concordant with protein levels. In fact, higher levels of full-length E-cadherin (120 KDa) were detectable in PC3 compared to DU145 2D-monolayers, and they were strongly up-regulated in 3D cell cultures compared to 2D-monolayers in both cell lines (Figure 4C). The analysis of the electrophoretic pattern also showed E-cadherin degradation fragments in both PC3 and DU145 2D-monolayers, consistent with cell junction disruption and, as a consequence, lower cell adhesion. A main fragment having an 80 kDa molecular weight was detected. These fragments were undetectable in cells grown in 3D-spheroids and 3D aggregates (Figure 4C). 

### 3.3. Expression of Mesenchymal Markers

Using immunofluorescence, we analyzed the expression of key EMT markers related to the acquisition of a mesenchymal phenotype. Confocal microscopy of 2D-monolayers revealed that N-cadherin was almost undetectable or expressed at very low levels in DU145 and PC3, respectively, showing that this mesenchymal marker is not detectable using a 2D cell culture. A different pattern of expression was observed for αSMA and vimentin immunoreactivity, that was strongly expressed in both cell lines (Figure 2 and Figure 3). The analysis of DU145 3D-spheroids showed that these mesenchymal markers were differentially expressed, other than αSMA, which was not influenced by 3D arrangement and therefore was evaluated only at the morphological level. Indeed, in DU145 cells grown in 3D-spheroids N-cadherin was detected in some few scattered cells, mostly located in the central region of the spheroid. Vimentin was expressed in all cells, although its immunoreactivity was more evident in the central region of the spheroids, similar to N-cadherin expression (Figure 2). Notably, the expression of vimentin and N-cadherin markers was more intense in the deep portion of the spheroids, while E-cadherin exhibited the opposite pattern of expression and was more intensely expressed in the outer region.

**Figure 5:**
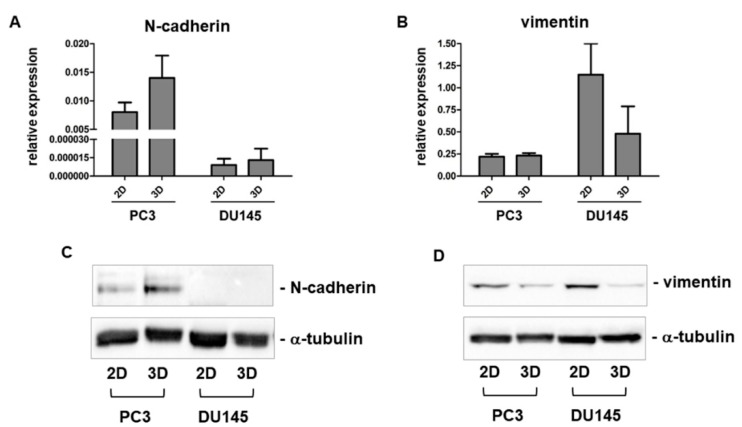
N-cadherin and vimentin mRNA and protein levels. Bar graphs showing N-cadherin (**A**) and vimentin (**B**) mRNA levels in PC3 and DU145 cells grown in 2D-monolayers and 3D cell cultures. Data are means ± SD of duplicate samples. Representative Western blot analysis showing N-cadherin (**C**) and vimentin (**D**) protein levels in whole cell lysates obtained from PC3 and DU145 cells cultured in both experimental conditions.

The expression of mesenchymal markers was also analyzed at the mRNA and protein level. According to the morphological analysis, N-cadherin gene expression was higher in PC3 cells while it was very low in DU145 cells cultured in 2D-monolayers (Figure 5A). While its expression was similar in DU145 cultures in 2D and 3D, N-cadherin gene expression was stimulated by 3D arrangement in PC3 cells (Figure 5A). Vimentin showed the opposite gene expression pattern, since it was similar in 2D and 3D cell cultures in PC3 cells, while expressed at a lower extent in DU145 3D compared to 2D cell cultures (Figure 5C). Since mRNA levels are not predictive of protein levels, due to translational and posttranslational regulations, N-cadherin and vimentin protein levels were analyzed. Higher N-cadherin protein levels were detected in PC3 3D-spheroids, consistent with gene expression, while they were undetectable in 2D and 3D cell cultures containing DU145 cells (Figure 5B). Vimentin protein levels were expressed at a lower extent in DU145 cells, similarly to mRNA, compared to PC3 cells. However, in both cell lines, vimentin was downregulated by 3D arrangement (Figure 5D).

**Figure 6:**
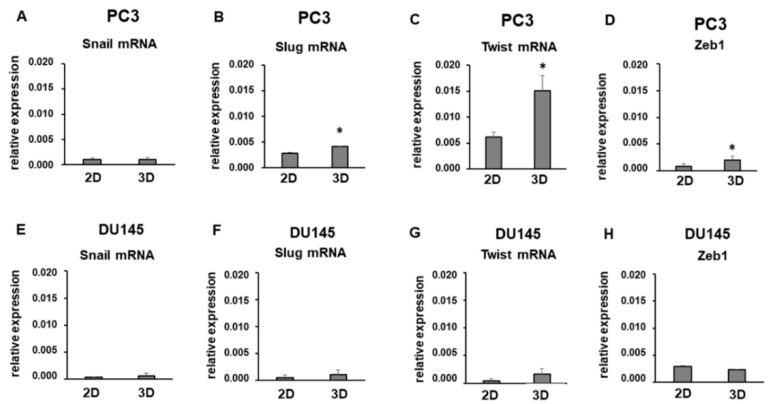
Snail, Slug, Twist and Zeb1 mRNA levels. Bar graphs showing Snail (**A**) and (**E**), Slug (**B**) and (**F**), Twist (**C**) and (**G**) and Zeb1 (**D**) and (**H**) mRNA levels in PC3 and DU145 cells grown in 2D-monolayers and 3D cell cultures. Data are means ± SD of duplicate samples. * *p* < 0.05 vs 2D-monolayer.

**Figure 7:**
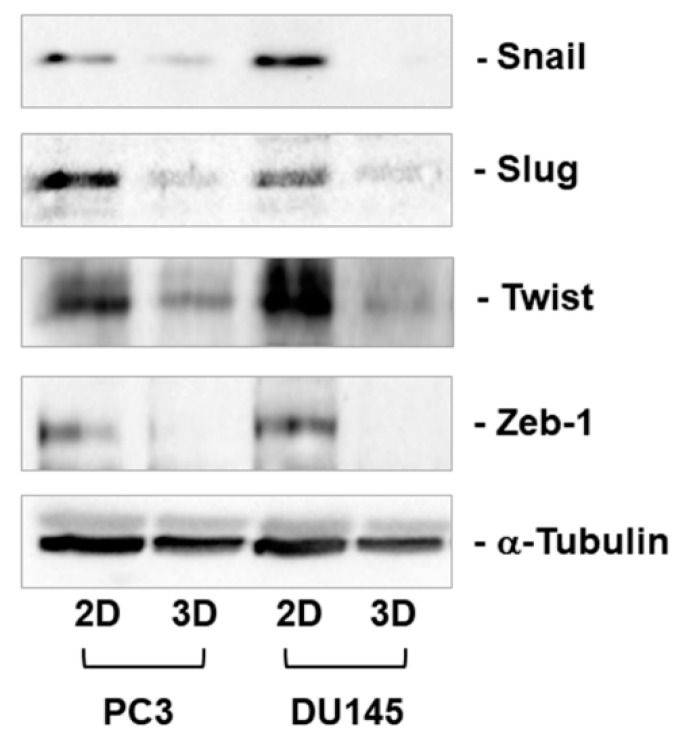
Snail, Slug, Twist, and Zeb1 protein levels. Representative Western blot analysis showing Snail, Slug, Twist, and Zeb1 protein levels in whole cell lysates obtained from PC3 and DU145 cells cultured in 2D-monolayers and 3D cell cultures.

Among EMT markers, EMT transcriptional regulators Snail, Slug, Twist, and Zeb1 play a key role. We analyzed their expression at the mRNA and protein levels, as shown in Figure 6 and Figure 7. Snail mRNA levels were similarly expressed in PC3 and DU145 2D-monolayers and 3D cell cultures (Figure 6A,E). Slug and Twist were expressed at a higher extent in PC3 compared to DU145 cells (Figure 6B,C,F,G), and they tended to increase in 3D-cell cultures, showing a significant up-regulation in PC3 3D-spheroids compared to 2D-monolayers (*p* < 0.05) (Figure 6B,C). Similar Zeb1 mRNA were detected in PC3 and DU145 cells, resulting in significantly increased PC3 3D aggregates compared to 2D-monolayers (*p* < 0.05) (Figure 6D,H). Gene expression was not confirmed by Western blot analysis, revealing that these EMT markers undergo important translational and posttranslational regulation. In fact, Snail, Slug, Twist, and Zeb1 were strongly down-regulated in 3D cell cultures compared to 2D-monolayers (Figure 7). Interestingly, low or undetectable expression of these EMT markers was consistent with the high expression of E-cadherin under both experimental conditions.

### 3.4. CD44 Gene Expression

**Figure 8:**
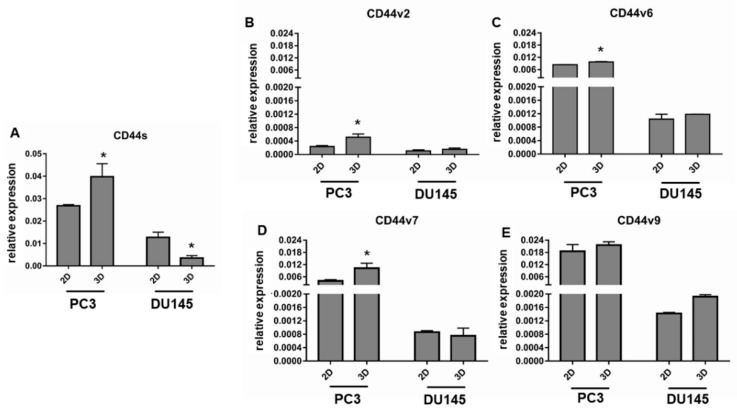
CD44s and variants gene expression. Bar graphs showing CD44s (**A**), CD44v2 (**B**), CD44v6 (**C**), CD44v7 (**D**) and CD44v9 (**E**) gene expression in PC3 and DU145 cells grown in 2D-monolayers and 3D cell cultures. Data are means ± SD of duplicate samples. * *p* < 0.05 vs 2D-monolayer.

**Figure 9:**
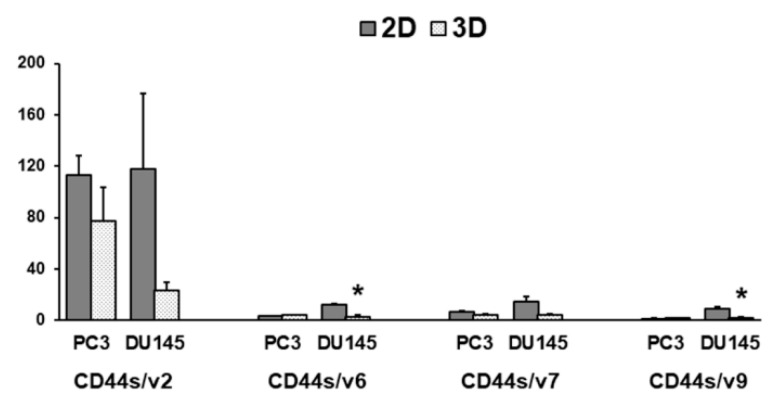
CD44s/CD44 variant in PC3 and DU145 cells. Bar graphs showing the CD44s/CD44 variant in PC3 and DU145 cells grown in 2D-monolayers and 3D cell cultures. Data are means ± SD of duplicate samples. * *p* < 0.05 vs 2D-monolayer.

Gene expression for CD44s and CD44 v2, v6, v7, and v9 variants was investigated by real-time PCR, showing that they are expressed at a higher extent in PC3 compared to DU145 cells. If we analyzed the effect of 3D arrangement, CD44s and variants were up-regulated in PC3 3D-spheroids compared to 2D-monolayers (Figure 8). The difference was statistically significant for CD44s, v2, v6, v7 (*p* < 0.05) (Figure 8A–D). CD44s and CD44 variants were expressed in DU145 cells at a lower extent, compared to PC3 cells, but only CD44s gene expression was affected by 3D arrangement, despite undergoing an opposite regulation compared to PC3 cells. In fact, CD44s was significantly down-regulated in DU145 spheroids compared to 2D-monolayers (*p* < 0.05) (Figure 8A), whilst CD44 isoform expression was not influenced by 3D arrangement (Figure 8B–E). To understand the functional role of CD44s and variant expression, the CD44s to CD44v ratio could be calculated. CD44s/v2 was highly expressed in PC3 and DU145 2D-monolayers, and tended to decrease in 3D cell cultures (*p* = 0.054 for DU145 cells) (Figure 9). CD44s/v6, CD44s/v7 and CD44/v9 were nor significantly affected by 3D arrangement in PC3 cells. By contrast, they were down-regulated in DU145 3D-spheroids (*p* < 0.05, *p* ns, *p* < 0.05, respectively) (Figure 9). 

## 4. Discussion

The loss of epithelial phenotype and cell-cell adhesion driven by the down-regulation of E-cadherin, a well characterized transmembrane protein of adherens cell junctions expressed in differentiated and polarized epithelial cells, is the key early event during EMT, and it is considered an essential prerequisite for carcinoma progression to metastatic disease [4]. However, tumors are heterogeneous and cancer cells may undergo only a partial transition that enhances invasion, while still retaining certain epithelial characteristics such as E-cadherin expression, leading to the observation of cells displaying an “intermediate” phenotype. Indeed, E-cadherin loss does not always correlate with invasion [38]. 

Some heterogeneity in E-cadherin expression has been previously described in pancreatic adenocarcinoma [31,39] as well as in PCa, showing variable E-cadherin expression in metastatic tissues compared to primary tumor tissues [15,16,17,18,19,20], thus increasing the relevance of studies aimed at characterizing the phenotype of PCa cells in relation to the expression of EMT markers, and especially of E-cadherin, in order to better understand PCa development and progression.

Since E-cadherin is considered a pivotal determinant in the EMT mechanism [4,5], we analyzed this EMT “epithelial” marker at the morphological and molecular level. Immunofluorescence analyses show that both PC3 and DU145 cells grown in 2D-monolayers are characterized by an intense E-cadherin immunoreactivity at the cell-cell boundary, suggesting that PC3 and DU145 cells have an epithelial phenotype. A similar E-cadherin immunoreactivity was detected in DU145 3D-spheroids, suggesting that the adherent junctions are retained by PCa cells under both experimental conditions. Gene expression analysis showed that E-cadherin mRNA levels tend to increase in PC3 cells and are significantly up-regulated in DU145 cells grown in 3D cell cultures. Since this pattern of expression parallels E-cadherin protein levels, cells grown in 3D-spheroids may have stronger cell adhesion, pointing to a possible transcriptional regulation of E-cadherin level in PCa cells. The significant up-regulation of E-cadherin mRNA levels in DU145 3D-spheroids compared to 2D-monolayers is consistent with the observation of more compact spheroids compared to PC3 cells, forming more irregular and loose cell aggregates, as previously described [33]. The analysis of the E-cadherin electrophoretic pattern revealed protein fragments detectable only in 2D-monolayer cell lysates, indicating a post-translational modification of this protein mediated by proteolytic cleavage. Partial E-cadherin degradation in PCa cells grown in 2D-monolayers could likely induce a reduced cell-cell adhesion, compared to cells grown in 3D-spheroids.

E-cadherin cleavage has been linked to the malignant progression of adenocarcinomas, including prostate cancer [40,41]. We observed 80, 65, and 55 kDa cleavage fragments of E-cadherin. The 80 kDa fragment was detected in the media of mouse and human mammary tumor cells [42] and described in prostate cancer patients [43].

The degradation and functional loss of E-cadherin are described as key molecular alterations that occur during tumor progression and are mediated by a variety of mechanisms, including transcriptional repression, epigenetic silencing, inactivating mutation, enhanced degradation, endocytosis, and proteolytic cleavage [44,45]. The 80 kDa proteolytic fragment could be generated by MMP activity in cell culture systems [46,47], pointing to the involvement of MMP in proteolytic cleavage events leading to E-cadherin inactivation and resulting in the disruption of cell-cell adhesion and the promotion of the malignant progression of prostate epithelial cells. 

Here we show that full length E-cadherin is up-regulated in 3D-spheroids. A similar situation was previously described in pancreatic adenocarcinoma cells [31], indicating a stronger cohesion by linking cells together and ensuring tissue integrity during collective cell migration [48]. This hypothesis is supported by the observation that the more intense E-cadherin immunoreactivity is evident at the periphery of 3D-spheroids corresponding to the leading edge of migrating epithelial sheets where the front cells retain intact cell–cell junctions to mechanically hold the cells together [49]. 

During EMT the “cadherin switch” is described, leading to the expression of N-cadherin, the transmembrane protein of adherens junctions in mesenchymal cells. Our data show that N-cadherin immunoreactivity was almost undetectable in DU145 and PC3 2D-monolayers, and seemed more evident in DU145 3D-spheroids. In fact, some few scattered cells expressing N-cadherin were detected in DU145 3D-spheroids, that, interestingly, were located in the central region of the spheroid where E-cadherin is less evident. This observation confirms that a cadherin switch occurred in PCa cells, and that this is more evident in PCa cells grown in 3D-spheroids. The expression of N-cadherin confirms the EMT-related phenotype of PCa cells. However, N-cadherin is less effective in forming cell junctions compared to E-cadherin, and its higher expression in PC3 cells is consistent with a “more mesenchymal phenotype” of these cells, compared to DU145, and with the irregular morphology of PC3 spheroids. The molecular analysis of N-cadherin in PC3 suggests that N-cadherin gene and protein expression is affected by 3D-spheroids, showing a higher expression of this EMT marker. The mesenchymal markers αSMA and vimentin were strongly expressed in PC3 and DU145 cells grown either in 2D or 3D cell cultures. Interestingly, vimentin immunoreactivity was more evident in the central region of DU145 spheroids, similarly to N-cadherin, where E-cadherin is less evident. Conversely, E-cadherin immunoreactivity is more intense in the outer region of 3D-spheroids, offering a higher cell adhesion. This finding seems to confirm the hypothesis that peripheral cells in PCa 3D-spheroids exhibit the properties of a leading edge invading surrounding tissues, where a more evident epithelial phenotype is functional to favor a collective migration and invasion of cancer cells. Tumors might utilize this particular strategy of migration to permit the movement of carcinoma cells that have not lost their well-differentiated epithelial morphology.

E-cadherin expression can be regulated through a combination of genetic, epigenetic, transcriptional and post-transcriptional mechanisms. Zinc finger family members Snail and Slug, the basic helix-loop-helix factor Twist, and the handed zinc factors Zeb1 act as major transcriptional repressors of E-cadherin [50].

Snail can be considered as one of the master EMT regulators and is a promoter of metastasis by inducing the expression of mesenchymal markers. Snail has also been implicated in cancer cell survival, cell cycle regulation, apoptosis evasion, cell adhesion, neuroendocrine differentiation, and chemoresistance [50,51,52]. A correlation between high Snail expression in tumors and tumor aggressiveness and poor prognosis was described [12,53,54], and Snail overexpression was detected in several cancer cells including PCa, where it has been suggested that it is up-regulated at early stages of PCa development [55].

Our results confirm the association of low E-cadherin with high Snail in cells grown in 2D-monolayers, showing a strong down-regulation of Snail in 3D-spheroids, paralleled by the increased E-cadherin expression.

Although Slug, Twist, and Zeb1 mRNA levels were significantly up-regulated in PC3 3D-spheroids, Slug, Twist and Zeb1 protein levels resulted strongly decreased, suggesting that their expression is likely dependent on mRNA stability, translational regulation, or posttranslational modification. 

Our results show that 3D arrangement affects PCa cell phenotype, especially in PC3 cells, and that PC3 and DU145 concomitantly express epithelial and mesenchymal markers having an intermediate phenotype. The expression of E-cadherin in PCa was previously investigated as a possible reliable marker for PCa. Its aberrant expression was detected, since loss of E-cadherin membrane staining was present in 14% of the morphologically normal prostates, more frequent in localized cancer samples (44% of cases), but not in metastasis (6% and 7% of cases) [56]. This is an interesting finding, pointing to an early and transient E-cadherin down-regulation favoring the detachment of cancer cells from the primary tumor, while metastatic cells retain high E-cadherin expression to favor their collective migration for tumor dissemination [19,57]. The concomitant cell-cell adhesion and expression of mesenchymal markers in PCa cells is a key feature of collective cell migration, and the possession of a partial EMT phenotype may be advantageous for metastatic progression by favoring both metastasis and metastatic recolonization [58].

According to this suggestion, we can hypothesize that transcription factors involved in EMT could be differently expressed in different moments during PCa progression, and the prognostic value of EMT markers should be carefully evaluated during cancer progression. However, the relationships between EMT transcription factors and target genes such as E-cadherin are likely to be more complex in vivo than those identified in cultured cells. A comparison of biological properties and behavior of PCa cells cultured in 2D-monolayers or 3D-spheroids was previously described by [34] on 29 prostate cancer cell lines, including PC3 and DU145 cells. That study described spheroid morphology and behavior, especially in relation to EMT and spheroid invasive potential. However, the results, although showing differences in 2D and 3D cell cultures, are not comparable with our data. In fact, 3D-spheroids were cultured in different experimental conditions using Matrigel, representing an important stimulus-inducing differentiation program different than in cells cultured without any interaction with ECM components. 

Cells undergoing EMT express biomarkers such as CD44, recognized as a cancer stem cell marker. CD44 is a widely expressed adhesion molecule, which contributes to cell-cell and cell-matrix adhesion, cell growth, differentiation and trafficking [59]. Alternative splicing of the gene encoding for CD44 produces CD44 standard (CD44s) and CD44 variant (CD44v) forms numbered from v1 to v10, some of which have been found to increase in tumor progression and metastasis [60,61]. It has been reported that a CD44 isoform switches from a variant form to a standard form during the EMT process and that CD44s has been correlated to a more mesenchymal phenotype, while the various CD44v forms are related to increased cell adhesion [35,36,62,63,64]. 

Several studies have described dysregulated CD44 expression in the majority of human cancers, including prostate cancer, and until now only few studies have explored the CD44 variants expressed in PCa, providing controversial results, and the functional role of CD44s and CD44v is not fully understood yet. For instance, CD44s together with an overexpression of all variants generated by alternative splicing were detected in metastatic PCa patients, compared to the benign hypertrophy controls [65], but it was also suggested that CD44s may exert an inhibitory effect on invasion and migration, and that divergent effects could depend upon the specific tumor environment [66]. To the best of our knowledge, this work is the first study investigating CD44s and variants in PCa cells grown in 3D-cell cultures, and the first study analyzing CD44 in relation to EMT markers, contributing to the comprehension of the expression of CD44 in PCa.

We detected higher CD44s mRNA levels in PC3 cells grown in 2D-monolayers compared to DU145, suggesting that PC3 have a more mesenchymal phenotype compared to DU145 cells. This gradient of EMT is consistent with higher N-cadherin mRNA levels in PC3 compared to DU145 cells. EMT has been linked to stem cell phenotype [35,36,37], and EMT results in the acquisition of stem cell-like properties, including slow proliferation and self-renewal potential [21,67,68]. Moreover, an important role was described for N-cadherin, acting as a positive regulator of metastatic abilities of PCa cells by modulating EMT and stemness properties via the receptor tyrosine kinase ErbB signaling [69]. Interestingly, 3D arrangement differently and significantly influenced PCa cell phenotype, inducing an opposite effect, that is a significant up-regulation in PC3 and a down-regulation in DU145 cells of CD44s. This result leads to the hypothesis that the 3D arrangement favors the acquisition of the mesenchymal characteristics of PC3, while favoring a more epithelial-like phenotype in DU145 cells. 

This idea was confirmed by the analysis of CD44s/CD44v6-7 and v9 ratios, which was useful for understanding the possible functional role of CD44s and CD44v. Indeed, we found a significant decrease of this ratio in DU145 3D-spheroids compared to 2D-monolayers. 

The expression of CD44 variants on the surface of tumor cells was related to different PCa characteristics and seems to be associated with invasive and metastatic behavior. Higher expression levels of CD44v2 were detected in patients with a better survival rate, and a lower expression of CD44v2 correlated with a 4 times greater risk of biochemical recurrence, suggesting CD44v2 has a role as a prognostic marker for PCa [66]. CD44v6 is a marker able to differentiate epithelial from mesenchymal prostate cancer cells [62,64]. It was also recently shown that CD44v6 expression is necessary for anchorage independent growth and that high expression is associated with a poor outcome in prostate cancer patients [70]. Other studies in lung, colon, and breast cancer confirm that higher CD44v6 expression is a negative prognostic indicator [71,72,73].

Here we have shown that also CD44 v2, v6, v7n and v9 resulted in differently expressed in PC3 and DU145 cells. We detected higher expression in PC3 compared to DU145 monolayers, and a significant up-regulation in PC3 3D aggregates compared to 2D-monolayers. According to the proposed roles for CD44 variants, our data suggest that PC3 cells could exhibit a more mesenchymal phenotype compared to DU145, and, very likely, a more malignant behavior. Conversely, 3D-arrangement elicits a different CD44s/v ratio in PC3 and DU145 cells, allowing the detection of a different behavior. Since CD44s and CD44v are related, respectively, to a more mesenchymal or epithelial phenotype, our results suggest that DU145 cells have a more epithelial phenotype, compared to PC3 cells, consistent with the stronger cell adhesion observed under the microscope.

The different phenotype, based on CD44 gene expression in PC3 and DU145 cells, was confirmed by a different response to IL-4, a regulator of basal-like cells showing characteristics of tumor-initiating cells by inducing expression of CD44. In fact, it was reported that IL-4 administration strongly induces CD44 expression in PC3, but not in DU145 cells [74].

## 5. Conclusions

Our study provides a comprehensive analysis of the main epithelial and mesenchymal EMT markers. Considered as a whole, our data could contribute to clarifying the expression of EMT markers in PCa cells and the role of EMT in PCa progression, providing additional evidence that PCa cells express EMT markers, and pointing to important differences in the phenotype of PCa cells grown in 3D cell cultures. Although PC3 formed 3D aggregates containing cells loosely apposed and their morphological characterization was not possible, PC3 grown in 3D aggregates were linked by cell junctions; therefore, a cell-cell interaction induced by the 3D arrangement can be effective in influencing cell phenotype, as confirmed by the molecular results, showing evident differences in PC3 2D-monolayers and 3D aggregates. Our data show that metastatic PC3 and DU145 PCa cells grown in 3D cell cultures could better reveal their morpho-functional characteristics and a gradient of EMT, representing a good experimental model useful for defining and recognizing cell phenotype in relation to EMT and CD44 expression. Indeed, the important morphological and molecular differences in cells grown in 2D-monolayers or in 3D cell cultures allow the identification of EMT-related markers differently expressed in 2D and 3D cell cultures, in order to plan future studies focused on the analysis of specific mechanisms underlying their roles in the complex EMT process. Moreover, they strongly support the use of 3D-spheroids as a more suitable experimental model to mimic PCa cell behavior in vitro, allowing us to distinguish and characterize different PCa cells, in order to understand and predict their behavior, thus adding new knowledge to PCa biology and progression.

Since, to the best of our knowledge, this work is the first study investigating EMT markers and CD44 variants in the same experimental conditions, and EMT has been linked to stem cell phenotype, we feel that our results could contribute to understand PCa cells’ biology and behavior.

## Figures and Tables

**Table 1:** List of primer used for gene expression analysis.

Gene	Primer Forward	Primer Reverse
GAPDH	CCCTTCATTGACCTCAACTACATG	TGGGATTTCCATTGATGACAAGC
E-cadherin	GAACGCATTGCCACATACAC	GAATTCGGGCTTGTTGTCAT
N-cadherin	CCTGAGGGATCAAAGCCTGGAAC	TTGGAGCCTGAGACACGATTCTG
Snail	CTTCCAGCAGCCCTACGAC	CGGTGGGGTTGAGGATCT
Slug	TGTTTGCAAGATCTGCGGC	TGCAGTCAGGGCAAGAAAAA
Twist	AGCAAGATTCAGACCCTCAAGCT	CCTGGTAGAGGAAGTCGATGTACCT
Zeb1	GAAAGTCATCCAGCCAAATGG	ACTTGGTTCTCAGCTTGGGGAATCA
CD44s	GGAGCAGCACTTCAGGAGGTTAC	GGAATGTGTCTTGGTCTCTGGTAGC
CD44v2	ATCACCGACAGCACAGACAGAAT	AACCATGAAAACCAATCCCAGG
CD44v6	CCAGGCAACTCCTAGTAGTACAACG	CGAATGGGAGTCTTCTTTGGGT
CD44v7	GCCTCAGCTCATACCAGCCATC	TCCTTCTTCCTGCTTGATGACCT
CD44v9	AGCAGAGTAATTCTCAGAGC	TGATGTCAGAGTAGAAGTTGTT

## Supplementary Materials

The supplementary materials are available online at http://www.mdpi.com/2073-4409/8/2/143/s1.
