# Supplementary material for: Epithelial-To-Mesenchymal Transition Markers and CD44 Isoforms Are Differently Expressed in 2D and 3D Cell Cultures of Prostate Cancer Cells
Source: Cells. 2019 Feb 11;8(2):143. doi: 10.3390/cells8020143 (PMC6406374; doi:10.3390/cells8020143)
Supplement: Supplementary file 1 [file cells-08-00143-s001.pdf]

Article

Epithelial-To-Mesenchymal Transition Markers and CD44 Isoforms Are Differently Expressed in 2D and 3D Cell Cultures of Prostate Cancer Cells

Fabrizio Fontana [1,†], Michela Raimondi [1,†], Monica Marzagalli [1], Michele Sommariva [2], Patrizia Limonta [1,‡] and Nicoletta Gagliano [2,‡,*]

Supplementary Materials:

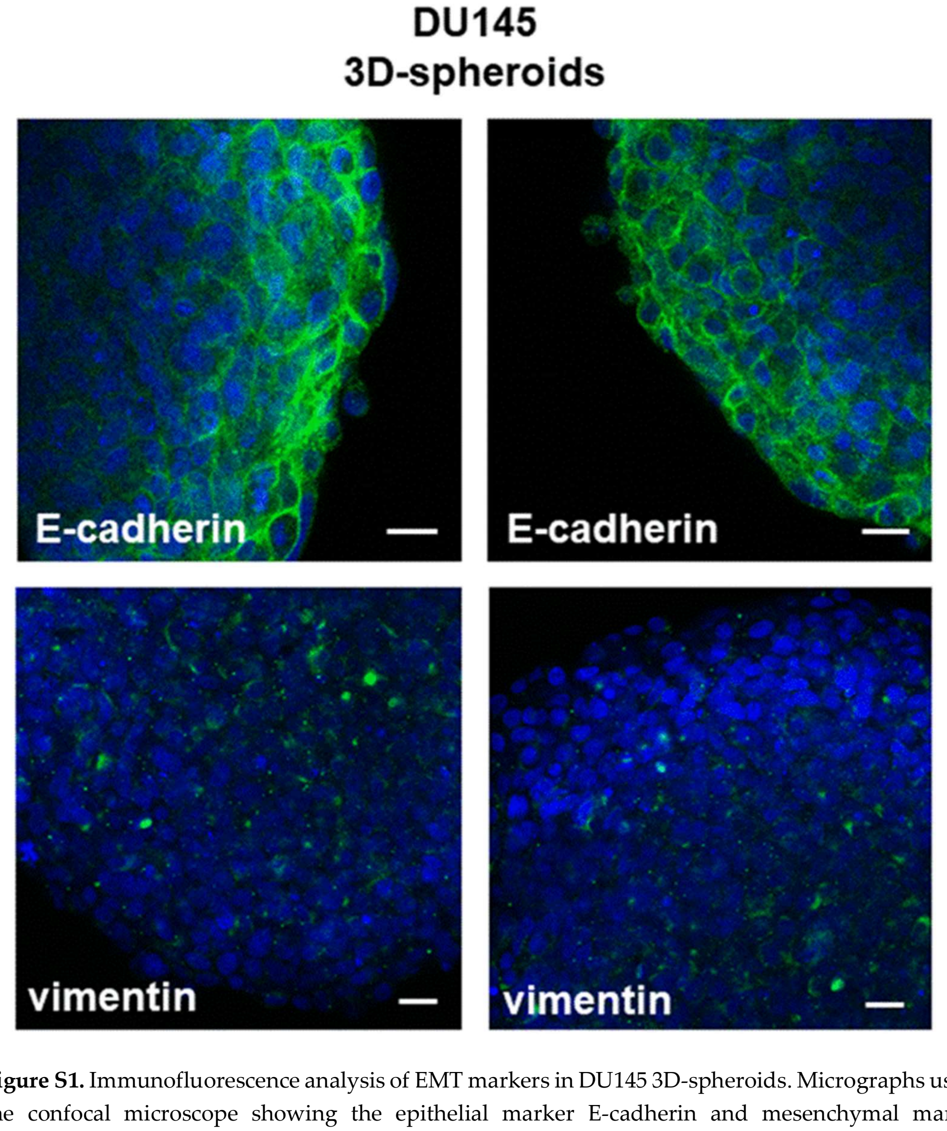

Figure S1. Immunofluorescence analysis of EMT markers in DU145 3D-spheroids. Micrographs using the confocal microscope showing the epithelial marker E-cadherin and mesenchymal marker vimentin (green) in DU145 cells grown in in 3D-spheroids. Immunoreactivity for E-cadherin is more intense in the periphery of the spheroid, whilst vimentin is mostly expressed in the deep regions of the spheroid. Original magnification: 40x. Bar: 20 μm. Blue: DAPI.
